# Synthetic Archaeosome Vaccines Containing Triglycosylarchaeols Can Provide Additive and Long-Lasting Immune Responses That Are Enhanced by Archaetidylserine

**DOI:** 10.1155/2012/513231

**Published:** 2012-09-30

**Authors:** G. Dennis Sprott, Angela Yeung, Chantal J. Dicaire, Siu H. Yu, Dennis M. Whitfield

**Affiliations:** Institute for Biological Sciences, National Research Council of Canada, 100 Sussex Drive, Ottawa, ON, Canada K1A 0R6

## Abstract

The relation between archaeal lipid structures and their activity as adjuvants may be defined and explored by synthesizing novel head groups covalently linked to archaeol (2,3-diphytanyl-sn-glycerol). Saturated archaeol, that is suitably stable as a precursor for chemical synthesis, was obtained in high yield from *Halobacterium salinarum*. Archaeosomes consisting of the various combinations of synthesized lipids, with antigen entrapped, were used to immunize mice and subsequently determine CD8^+^ and CD4^+^-T cell immune responses. Addition of 45 mol% of the glycolipids gentiotriosylarchaeol, mannotriosylarchaeol or maltotriosylarchaeol to an archaetidylglycerophosphate-O-methyl archaeosome, significantly enhanced the CD8^+^ T cell response to antigen, but diminished the antibody titres in peripheral blood. Archaeosomes consisting of all three triglycosyl archaeols combined with archaetidylglycerophosphate-O-methyl (15/15/15/55 mol%) resulted in approximately additive CD8^+^ T cell responses and also an antibody response not significantly different from the archaetidylglycerophosphate-O-methyl alone. Synthetic archaetidylserine played a role to further enhance the CD8^+^ T cell response where the optimum content was 20–30 mol%. Vaccines giving best protection against solid tumor growth corresponded to the archaeosome adjuvant composition that gave highest immune activity in immunized mice.

## 1. Introduction

The total polar lipids extracted from various archaea hydrate to form liposomes (archaeosomes [1]), that were developed initially to improve the drug delivery application of conventional liposomes [2–4]. These total polar lipid archaeosomes were found subsequently to have an enhanced ability over conventional liposomes to serve as adjuvants, that promoted not only the antibody response to an entrapped protein antigen [5] but also the CD8^+^ T cell response [6]. One mode of action could be correlated to an enhanced phagocytosis of archaeosomes compared to liposomes by various phagocytic cells [7]. This led to the observation that total polar lipids from various archaea, with their species-specific lipid structures, formed archaeosomes differing in receptor-mediated endocytosis and adjuvanticity [8]. 

Recently archaeol has been isolated from hydrolysed polar lipid extracts of *Halobacterium salinarum *to use as the lipid precursor to chemically synthesize various polar lipids, including glycolipids [9, 10]. The lipids so generated are described as synthetic or more precisely as semisynthetic, because the lipid moiety with specific archaeal sn-2,3 and R-methyl group stereochemistry is of biological origin, whereas a polar head group may be conjugated to the free sn-1 hydroxyl of the glycerol backbone to give a new polar lipid structure. In this way a chemically-defined, synthetic archaeosome could in theory be optimized for each application. Feasibility was demonstrated by synthesizing a series of diglycosylarchaeols and testing their interactions with antigen-presenting cells to produce immune responses *in vivo* [9]. 

The long-lasting CD8^+^ T cell memory responses that are generally thought to be required for protection in intracellular pathogen and cancer vaccines are induced by certain total polar lipid archaeosomes and have been correlated to those archaeosomes having a high proportion of membrane-spanning caldarchaeol (tetraether) lipids [6, 11]. In this study we explore whether synthetic archaeosome adjuvants that are based on the archaeol lipids without caldarchaeols, can provide such long-term responses. Further, we explore if synthetic archaetidylserine, previously found to interact positively with the phosphatidylserine receptor of antigen-presenting cells [8, 12], can augment the adjuvant activity of synthetic glycolipid archaeosomes.

## 2. Materials and Methods

### 2.1. Growth of Archaea

*Halobacterium salinarum* (ATCC 33170) was grown aerobically at 37°C in a medium modified to be an all nonanimal origin medium consisting of: 15 g/L Phytone peptone UF (product 210931 from VWR International); 220 g/L NaCl; 6.5 g/L KCl; 10 g/L MgSO_4_·7H_2_O; 10 mL of 0.2 g/100 mL CaCl_2_; 10 mL of 0.2 g/100 mL FeSO_4_. Growth of *Haloferax volcanii* (ATCC 29605) was in medium ATCC 974 at 30°C with NaCl content of 12.5% [13]. The antifoam agent used was MAZU DF 204 (BASF Canada). Biomass was grown in 20 L medium in a 28 L New Brunswick Scientific fermentor and harvested after 72 h growth. Lipids were extracted from the biomass with chloroform/methanol/water and the total polar lipids precipitated from the lipid extract with cold acetone [14].

### 2.2. Purification of Archaeol

Typically, 3.5 g of total polar lipid from *H. salinarum* was dissolved in 45 mL of chloroform/methanol (2 : 1, v/v) and 190 mL methanol added. This mixture was cooled to 0°C in an ice bath, and 10 mL acetyl chloride added drop-wise while being stirred magnetically. Hydrolysis was accomplished by refluxing at 62°C for 3 h. The mixture was cooled and the volume reduced by rotary evaporation to 100 mL. Upon transfer to a separatory funnel, 12 mL water and 100 mL petroleum ether was added. The mixture was mixed and allowed to separate. The top ether phase containing lipid was pooled with a second ether extraction, and evaporated.

The archaeol oil obtained above was further purified by silica gel column chromatography. The oil dissolved in chloroform/methanol (2 : 1, v/v) was loaded on a Silica gel 60 (Merck) column and archaeol eluted with pressure using hexane/t-butylmethylether/acetic acid (80/20/0.5, v/v/v). Collected fractions were tested for archaeol by mini thin-layer chromatography using the eluting solvent, and fractions containing pure archaeol pooled and dried. The yield of archaeol from total polar lipid ranged from 43 to 53%. Structural identity and purity of archaeol was confirmed by both NMR spectroscopy and electrospray ionization mass spectrometry.

### 2.3. Chemical Synthesis

**Figure 1:**
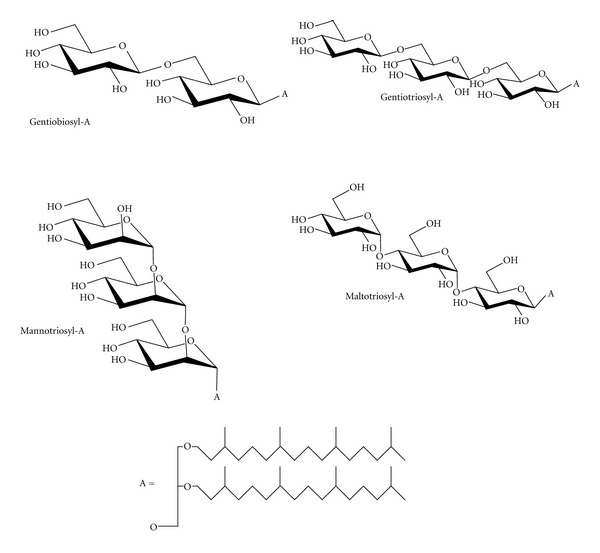
Semisynthetic glycoarchaeol structures showing head group details. “A” is the archaeol lipid precursor used for synthesis. gentiobiosyl-A (*β*-Glc_p_-(1→6)-*β*-Glc_p_-(1→O)-archaeol); Gentiotriosyl-A (*β*-Glc_p_-(1→6)-*β*-Glc_p_-(1→6)-*β*-Glc_p_-(1→O)-archaeol); mannotriosyl-A (*α*-Man_p_-(1→2)-*α*-Man_p_-(1→2)-*α*-Man_p_-(1→O)-archaeol); maltotriosyl-A (*α*-Glc_p_-(1→4)-*α*-Glc_p_-(1→4)-*β*-Glc_p_-(1→O)-archaeol).

Archaetidylethanolamine was synthesized according to [15]. Mannotriosylarchaeol, maltotriosylarchaeol, gentiobiosylarchaeol, and gentiotriosylarchaeol were synthesized according to our previous descriptions [10, 16] and structural details are shown in Figure 1. Synthesis methods for archaetidylserine can be found in Supplementary Material available online at doi:10.1155/2012/513231.

### 2.4. Purification of PGP

Archaetidylglycerolphosphate-O-CH_3_ (PGP) was purified from the total polar lipids of *Haloferax volcanii* as described [13].

### 2.5. Archaeosome Vaccines

Archaeosomes were formed by hydrating 20–30 mg dried lipid at 40°C in 2 mL PBS buffer (10 mM sodium phosphate, 160 mM NaCl, pH 7.1) with ovalbumin Type VI (OVA, Sigma) as the test antigen dissolved at 10 mg/mL. Vesicle size was reduced to about 100–150 nm diameter by brief sonication in a sonic bath (Fisher Scientific), and OVA not entrapped was removed by centrifugation from 7 mL PBS followed by 2 washes (200,000 x g max for 90 min). Vesicle pellets were resuspended in 2–2.5 mL PBS and filter sterilized through 0.45 *μ*m Millipore filters. Sterile conditions and pyrogen-free water were used throughout.

Quantification of antigen loading was conducted by separating OVA from lipids using SDS polyacrylamide gel electrophoresis and densitometry as described [14]. Loading was based on *μ*g protein/mg salt corrected dry weight of lipid. Average diameters based on intensity were measured using a Malvern Nano Zetasizer with a He/Ne laser (Spectra Research Corp., Ontario, Canada).

### 2.6. Animal Trials

C57BL/6 female mice (6–8 weeks old) were immunized subcutaneously near the tail base with 0.1 mL vaccines containing the equivalent of 20 *μ*g OVA, often entrapped in archaeosomes of various compositions. A booster consisting of the same vaccine and route was given on week 3. All protocols and SOPs were approved by the NRC Animal Care Committee and conducted within the guidelines of the Canadian Council on Animal Care. 

### 2.7. Immune Responses

As a measure of the Th2 arm of CD4^+^ T cell adjuvant activity, IgG antibody raised in response to the antigen in the vaccine and collected in the sera of mice (5-6 mice/group) was quantified by Elisa according to a previous description [17]. The CD8^+^ T cell response was quantified by sacrificing duplicate mice/group to obtain their splenic cells. These splenic cells were assayed in triplicate for antigen-specific responses by standard Elispot and cytolytic T lymphocyte (CTL) methods [18].

### 2.8. Dendritic Cell (DC) Maturation Assay

Bone marrow was flushed from femurs and tibias of C57BL/6 mice to isolate DCs. Cells obtained were cultured in RPMI medium supplemented with 8% fetal calf serum (R8) (Thermo Scientific HyClone, UT, USA) and 5 ng/mL of granulocyte macrophage colony-stimulating factor (ID Labs, Inc., Ont., Canada) [19]. Nonadherent cells were removed on days 2 and 4 and supplied with fresh medium. Bone marrow DCs were harvested as the nonadherent cells on day 7. DC purity was greater than 90% based on flow cytometry of cells labeled with PE-Cy7 conjugated anti-CD11c mAb (BD Biosciences, Ont., Canada). To activate, on day 7 DCs (3 × 10^5^ cells/mL) were stimulated with 25 *μ*g of various antigen-free archaeosomes or 1 *μ*g *E. coli *lipopolysaccharide (LPS, Sigma-Aldrich, Ltd., Ont., Canada) per mL in 24-well plates for 24 h. Maturation was measured by the FITC-dextran (Sigma-Aldrich, Ltd., Ont., Canada) uptake assay using flow cytometry [20]. DCs were suspended in R8 medium and incubated with 1 mg/mL of FITC-dextran (Mr = 40 000) for 30 min at 4 or 37°C. After incubation, the cells were washed three times with ice cold 1% sodium azide in PBS. The quantitative uptake was calculated as the change in the Mean Fluorescence Index (MFI) between cell samples incubated at 37 and 4°C. 

### 2.9. EG.7 Solid Tumour Model

C57BL/6 mice were immunized at 0 and 3 weeks subcutaneously with archaeosomes containing 20 *μ*g OVA. A challenge consisting of 5 × 10^6^ EG.7 cells was introduced subcutaneously in the shaved lower dorsal region at either 4.5 weeks, or 14 weeks from the second immunization. Tumour progression was measured in two dimensions with a digital calliper, and values multiplied to give tumour sizes. When a tumour mass of 300 mm^2^ was reached the mouse was euthanized. 

### 2.10. Statistics

A comparison of means for animal data was conducted using student's *t*-test to determine significance at 95% confidence, and two tailed *P* values calculated using GraphPad Prism 5.

## 3. Results

### 3.1. Synthetic Glycosylarchaeols as Adjuvants

To prepare stable glycolipid archaeosome adjuvants from neutrally charged glycosylarchaeols it was necessary to include a charged lipid. This function may be served by a conventional ester-phospholipid such as phosphatidylglycerol. Although mice vaccinated with archaeosomes consisting of synthetic diglycosylarchaeols mixed with dipalmitoyl phosphatidylglycerol and antigen developed short-term CD8^+^ T cell mediated immune responses [9], longer-term responses were lost [15]. Consequently, we avoided conventional lipids in this study designed to evaluate the potential for long-term immunity from archaeol adjuvants, and chose instead an archaeol-based anionic lipid, PGP, purified from *H. volcanii*. The combination of glycosylarchaeols with PGP resulted in stable bilayers in the 100 nm average diameter range that entrapped the OVA antigen from 12–21 *μ*g protein/mg dry weight (Table 1).

**Figure 2:**
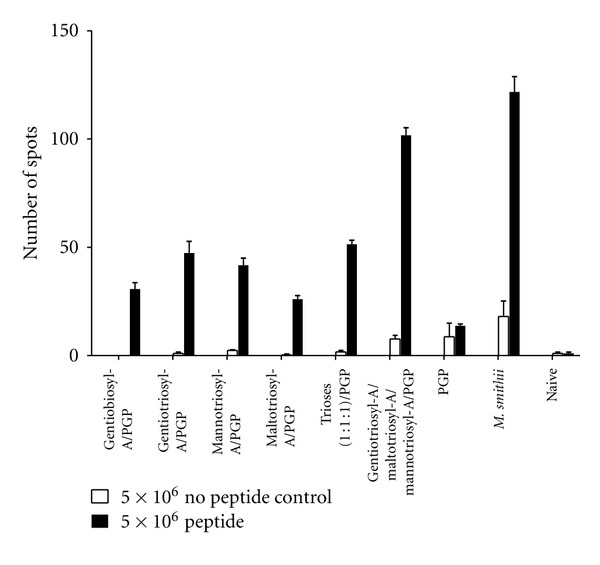
Antigen-specific CD8^+^ T cell activity in splenic cells of immunized mice as assayed by Elispot. Ratios of lipids in mol% for various compositions of archaeosomes were: di or triglycosylarchaeols/PGP (45/55), and gentiotriosyl-A/maltotriosyl-A/mannotriosyl-A/PGP (15/15/15/55), where “A” refers to archaeol. Trioses (1 : 1 : 1)/PGP refers to admixed triglycosyl-A/PGP vaccines, such that each contributed equal amounts of antigen. *M. smithii *represents OVA-loaded archaeosomes consisting of total polar lipids from* M. smithii*, as positive control. Mice were immunized subcutaneously at 0 and 3 weeks with OVA-loaded archaeosome adjuvants. Nonimmunized mice (naive) were included as negative controls. Spleens from duplicate mice were collected 5.5 weeks after first injection to determine the frequency (number of spots) of interferon-gamma (IFN-*γ*)-secreting splenic cells (spots) by enzyme-linked immunospot assay (Elispot). Omission of the major CD8 epitope of OVA (SIINFEKL) from the assay (no peptide control) was used to test for nonspecific responses. Means significantly different (*P* < 0.05) were gentiotriosyl-A/PGP versus maltotriosyl-A/PGP (*P* = 0.0204), mannotriosyl-A/PGP versus maltotriosyl-A/PGP (*P* = 0.0135), and maltotriosyl-A/PGP versus PGP (*P* = 0.0032). Those not significantly different were gentiotriosyl-A/PGP versus mannotriosyl-A/PGP (*P* = 0.4238), gentiotriosyl-A/PGP versus gentiobiosyl-A/PGP (*P* = 0.0550), mannotriosyl-A/PGP versus gentiobiosyl-A/PGP (*P* = 0.0677), and gentiotriosyl-A/maltotriosyl-A/mannotriosyl-A/PGP versus *M. smithii *(*P* = 0.0657).

First, we tested CD8^+^ T cell responses in immunized mice using glycosylarchaeol/PGP adjuvants in short-term experiments assayed 2.5 weeks from the booster immunization (Figure 2). Elispot assays confirmed gentiotriosylarchaeol to be a better adjuvant than gentiobiosylarchaeol. Although not highly significant for the data shown here (*P* = 0.055), in other trials the difference in means was characteristically *P* = 0.001. Further, both gentiotriosylarchaeol and mannotriosylarchaeol were significantly better adjuvants with PGP than was maltotriosylarchaeol. When all three triosylarchaeol vaccines were admixed in equal proportion prior to immunization the CD8 response was not greatly improved. However, a strikingly improved adjuvant activity, approaching the *M. smithii* total polar lipid positive control, was observed when the triglycosylarchaeols were incorporated into the same archaeosome preparation during hydration.

**Figure 3:**
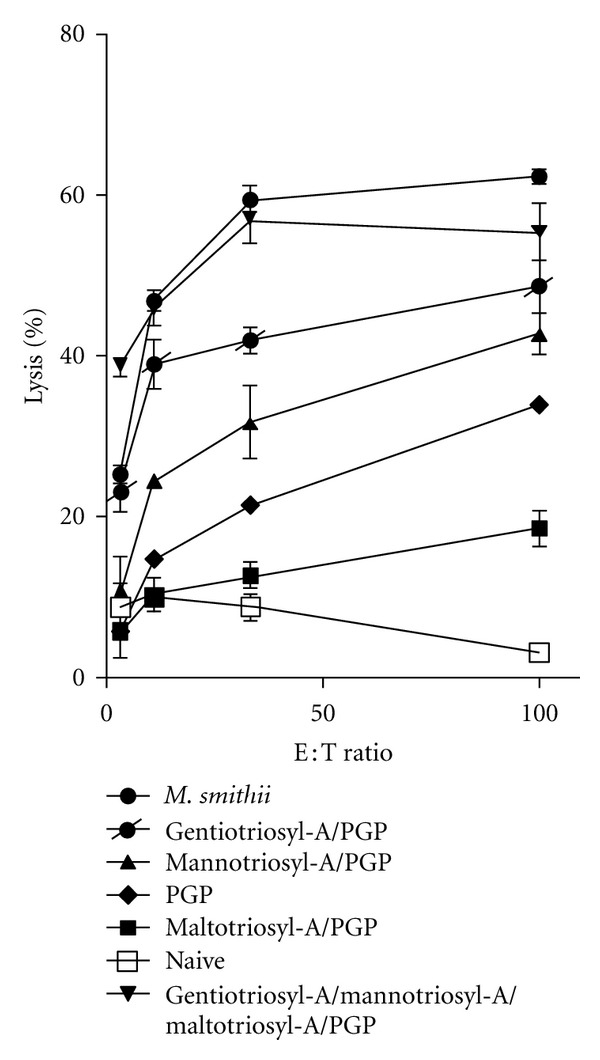
A cytotoxic T lymphocyte (CTL) lysis assay was used to assess the same populations of splenic cells as in Figure 1. The standard ^51^Cr assay was conducted using specific and nonspecific target cells (EG.7 and EL-4, resp.). The ratio of effector splenic cells to target cells is shown as the E : T ratio in the graph. Results shown are for EG.7 targets. EL-4 targets produced only low nonspecific responses (not shown).

CD8 responses in mice can also be measured by cytolytic T lymphocyte (CTL) assays that measure the ability of effector cells in the spleens of immunized mice to lyse an EG.7 target cell line expressing the dominant epitope (SIINFEKL) of OVA. In this assay (Figure 3) the same trends as found in Elispots occurred, although maltotriosylarchaeol/PGP was less effective as an adjuvant than pure PGP archaeosomes. The combined triosylarchaeols/PGP (45/55 mol%) again produced an adjuvant equivalent to the total polar lipid positive control. Because of these results, we omitted maltotriosylarchaeol from further studies, and continued with the combination of gentiotriosylarchaeol/mannotriosylarchaeol/PGP.

**Figure 4:**
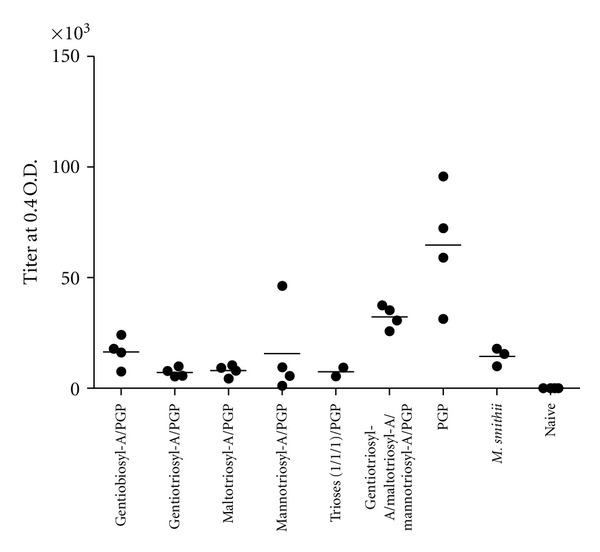
Antibody titres in sera of mice immunized with various archaeosome adjuvants. Peripheral blood was collected at 5.5 weeks, just prior to euthanizing mice for spleen removal (Figure 1). Each data point shown represents the titre in the serum from an individual mouse. Means were significantly higher for the OVA-PGP archaeosome vaccinated group (*P* < 0.05) compared to all groups except for Gentiotriosyl-A/Maltotriosyl-A/Mannotriosyl-A/PGP (*P* = 0.0560).

To evaluate the ability of archaeosome adjuvants to direct antigen via antigen-presenting cells through MHC class-II presentation to CD4^+^ T cells (see Figure 1 of [9]), we assayed anti OVA antibody titres in the peripheral blood of mice (Figure 4). Best titres were found for PGP archaeosomes, indicating that these archaeosomes favour an MHC-II route of antigen presentation versus MHC-I (as measured by CD8^+^ T cell responses). Antibody titres for PGP were significantly higher for all adjuvants except when compared to the combination of triosylarchaeols, which was not significantly different (*P* = 0.056).

### 3.2. Archaetidylserine (AS) and Archaetidylethanolamine (AE)

**Figure 5:**
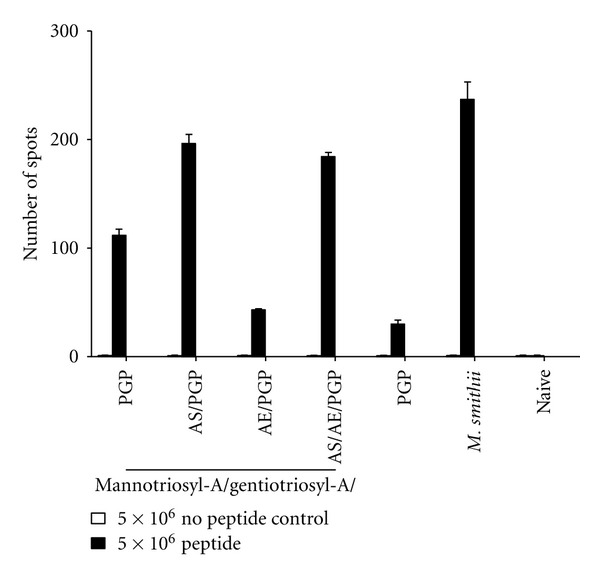
Elispot assay showing a relationship between adjuvant activity of glycosylarchaeol/PGP archaeosomes, archaetidylserine (AS), and archaetidylethanolamine (AE). Mol% compositions for OVA-archaeosome vaccines were mannotriosyl-A/gentiotriosyl-A/PGP (22.5/22.5/55), mannotriosyl-A/gentiotriosyl-A/AS/PGP (22.5/22.5/30/25), mannotriosyl-A/gentiotriosyl-A/AE/PGP (22.5/22.5/5/50), mannotriosyl-A/gentiotriosyl-A/AS/AE/PGP (22.5/22.5/30/5/20). Assays were conducted on splenic cells of mice 6 weeks post first immunization.

The phosphatidylserine receptor is implicated in promoting phagocytosis of apoptotic cell debris [21] and archaeosomes [12]. Further, both archaetidylserine and archaetidylethanolamine are potentially fusogenic lipids, based on the assumption of similar activity to their ester analogs [22], and fusion of internalized archaeosomes with the phagolysosome membrane is the mechanism proposed to export antigen from archaeosomes to the MHC-I pathway of antigen-presenting cells [12, 23]. Consequently, importance of AS or AE incorporated into the mannotriosylarchaeol/gentiotriosylarchaeol/PGP archaeosome was assessed in terms of adjuvanting CD8^+^ T cell responses (Figure 5). Addition of 30 mol% AS to the glycotriosylarchaeol/PGP adjuvant resulted in a significantly higher CD8 response (*P* = 0.0207) that was not significantly different than the positive control (*M. smithii*). AE combined with AS had little further influence on adjuvanticity. As in other mouse trials, incorporation of glycoarchaeols to PGP archaeosomes produced a much improved CD8^+^ T cell response. In contrast, anti OVA antibody titres in peripheral blood were not significantly higher upon inclusion of AS (data not shown).

**Figure 6:**
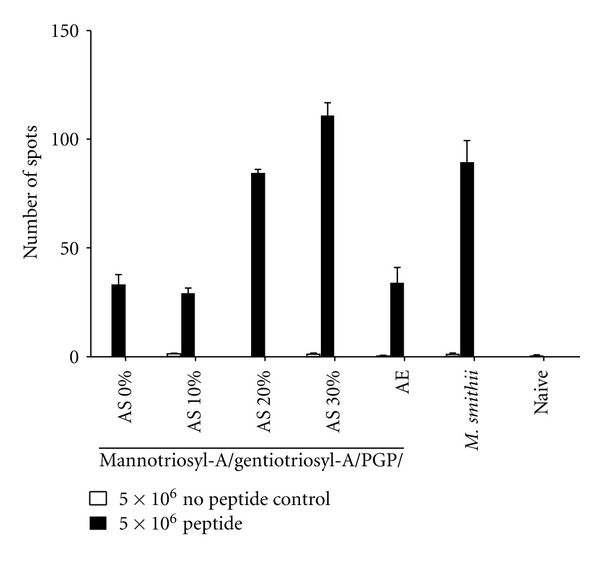
Elispot assay showing a relationship between mol% AS in a triglycosyl-A/PGP archaeosome and CD8 adjuvant activity. Mannotriosyl-A and gentiotriosyl-A were always 22.5 mol% each. AS was varied as shown at 0, 10, 20, and 30 mol%, with PGP making the remainder of each composition. For comparison, archaeosomes containing 5 mol% AE and *M. smithii* total polar lipid archaeosomes are included.

**Figure 7:**
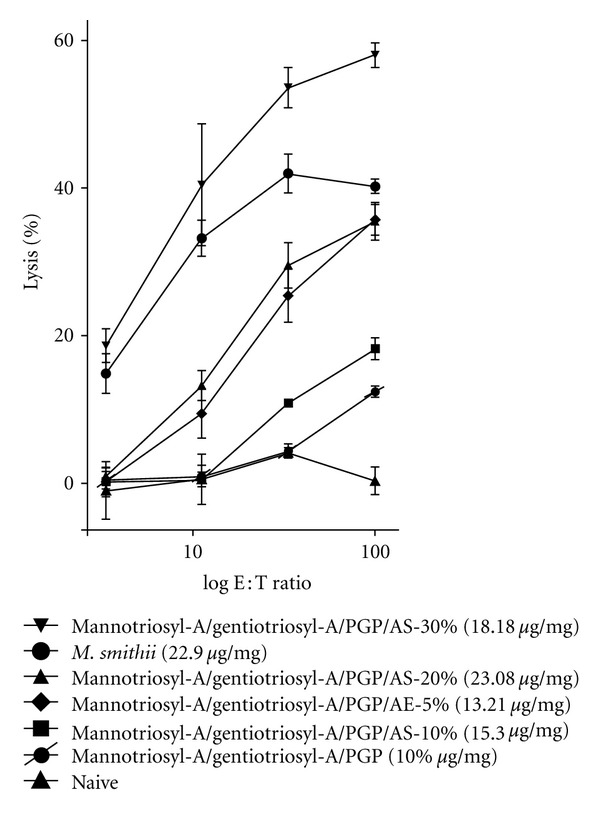
Cytotoxic T lymphocyte (CTL) lysis assay was used to assess the same populations of splenic cells as in Figure 6. Loadings of the OVA antigen are shown also in this figure. EL-4 control targets not expressing SIINFEKL gave <10% lysis in all cases (not shown).

To quantify the optimal amount of AS to adjuvant the CD8^+^ T cell mediated response, from 0 to 30 mol% AS was incorporated into the triglycosylarchaeol/PGP archaeosome. Elispot assays (Figure 6) showed little effect of 10% AS, with an optimal effect of >20–30 mol%. Archaeosomes could not be tested with >30 mol% AS because of instability. These findings were verified by CTL assays (Figure 7), that confirmed an adjuvant activity at 30 mol% AS to be somewhat higher than the positive control. As shown in Figure 5, the addition of AE to the adjuvant mix was rarely positive. 

### 3.3. Maturation of DCs

**Figure 8:**
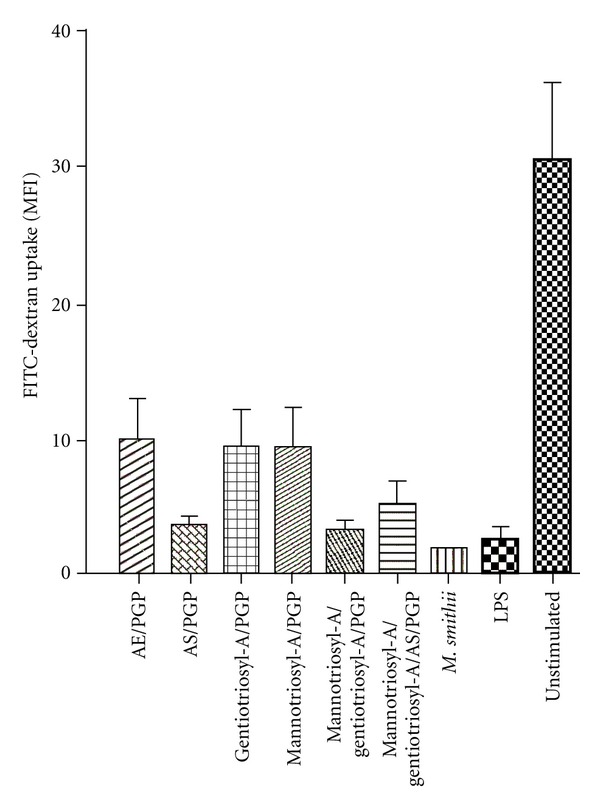
Maturation of dendritic cells (DCs) upon treatment with archaeosomes as measured by decrease of FITC-dextran uptake. Bone marrow DCs treated with archaeosomes *in vitro* were compared for their ability to take up FITC-dextran. The results depict the mean ΔMFI (37–4°C). Data represent means ± SD of triplicate cultures as indicated.

 Loss of ability to take up dextran was used to assess the extent of activation of DCs exposed *in vitro* to the various archaeosomes (lacking antigen) (Figure 8). LPS served as a positive control. Activation was similar for LPS, *M. smithii* archaeosomes, and the combination of gentiotriosylarchaeol/mannotriosylarchaeol/PGP with or without AS. Evidence for AS activation could be seen, however, by comparing AS/PGP (30/70 mol%) archaeosomes to either of AE/PGP (5/95 mol%), gentiotriosylarchaeol/PGP, or mannotriosylarchaeol/PGP archaeosomes.

### 3.4. Short and Long-Term Protective Immunity

**Figure 9:**
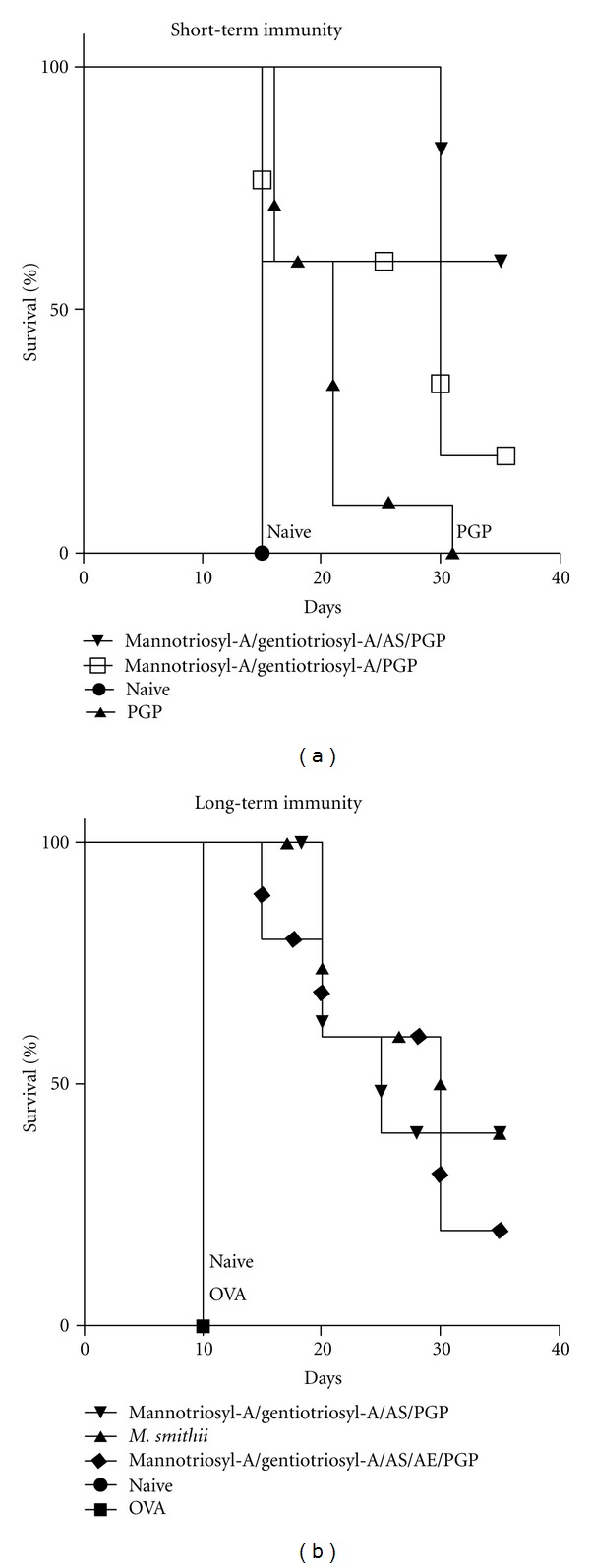
Protection of mice immunized with archaeosomes of various lipid compositions in a solid tumour model. Groups (*n* = 5) of unvaccinated mice (naive) or mice vaccinated with various OVA-archaeosomes were challenged with a subcutaneous injection of EG.7 cells (time zero) either 4.5 weeks (panel (a)) or 14 weeks (panel (b)) following their last vaccination.

*M. smithii* total polar lipid archaeosomes are capable of adjuvanting a CD8^+^ T cell response that is long-lasting and provides protection in a solid tumour model in mice [11, 18]. Here we compare immune responses to protection in mice immunized with the various synthetic archaeosomes-OVA. Short-term immunity was assessed in animals (*n* = 5) by challenge with EG.7 tumour cells 4.5 weeks after the second immunization (Figure 9(a)). Protection could be correlated to the CD8^+^ T cell immune responses achieved (see previous figures). Naive mice are considered unprotected and succumbed to tumour growth early. PGP-OVA archaeosomes showed only limited protection, with best protection achieved with mannotriosylarchaeol/gentiotriosylarchaeol/AS/PGP OVA-archaeosomes. Longer-term immunity was assessed by injection of EG.7 cells 14 weeks following the second immunization (Figure 9(b)). Nonimmunized naive mice and OVA immunizations (no adjuvant) showed no protection, whereas the optimized archaeosome gave protection similar to the positive control.

## 4. Discussion

A goal of this study was to define an archaeosome adjuvant composition suitable for human application through use of synthetic archaeol-based lipids. Past studies on the mechanism of archaeosomes made from the total polar lipids of various archaea have shown that adjuvant activity occurs at the level of the antigen-presenting dendritic and macrophages cells [19, 24]. Glycolipids in these total polar lipid mixtures may presumably serve as effective adjuvant ingredients as they can target specific receptors on antigen-presenting cells [9, 25, 26]. 

As glycolipids are uncharged, a stable bilayer does not form when attempts are made to prepare pure glycolipid-liposome based vaccines. This can be achieved, as is the case for natural polar lipids consisting of both glyco and phospholipids, by including phospholipids in the glycolipid formulation [9]. Because inclusion of nonarchaeal lipids such as dipalmitoyl phosphatidylglycerol into archaeosomes results in decline in longer-term CD8^+^ T cell mediated immune responses [15], we used the diacidic extreme halophile lipid, PGP, in our synthetic glycoarchaeol formulations.

A series of mannosylarchaeols synthesized to have from 1 to 5 sugar units, hydrated best and gave best adjuvant activity at 3 or 4 linear sugar units [16]. Similarly, we found gentiotriosylarchaeol to be a better adjuvant than gentiobiosylarchaeol. Further, the additive adjuvant effect obtained by inclusion of both gentiotriosylarchaeol and mannotriosylarchaeol suggests multiple positive interactions with receptors, to account for an observed increased activation of antigen-presenting dendritic cells (Figure 8). This additive effect of glycosylarchaeols required that the archaeosome preparation be hydrated with all lipids present, suggesting that the various head groups on the archaeosome surface were presented simultaneously to multiple receptors *in vivo*.

Archaetidylserine (AS) as a component of gentiotriosylarchaeol/mannotriosylarchaeol/PGP archaeosomes increased the CD8^+^ T cell immune response to entrapped antigen in a concentration dependent manner, without significantly enhancing the antibody response (Figures 6–7). *M. smithii* total polar lipid archaeosomes contain AS and their endocytosis has been linked to interaction with the phosphatidylserine receptor of antigen-presenting cells [12]. The pathway of cross-presentation of antigen carried in *M. smithii* archaeosomes occurs at the late phagolysosome stage [12] when calcium is internalized [27], suggesting that AS also contributes to membrane fusion promoted by calcium in analogy to phosphatidylserine [23]. Fusion of archaeosomes with the phagolysosome membrane would contribute to export of antigen to the cytosol and provide access to the MHC class-I presentation pathway. 

The longevity of CD8^+^ T cell memory induced by total polar lipid archaeosomes of *M. smithii* and *Thermoplasma acidophilum* is generally not found in archaeosomes prepared from total polar lipids of extreme halophiles, that lack caldarchaeols [6]. For this reason, it was proposed that long-term CD8^+^ T cell memory may require the presence of high proportions of caldarchaeol membrane-stabilizing lipids. In this study we found that protective CD8^+^ T cell memory responses could be induced in mice immunized with antigen-archaeosomes lacking caldarchaeols. This further indicated the importance of head group in lipid composition of an all archaeol-based adjuvant [9].

## 5. Conclusion

The immune response to antigen may be preferentially directed to either MHC-I (CD8) or MHC-II (CD4) presentations by selection of the head group(s) of an archaeol-based adjuvant. PGP archaeosomes direct antigen primarily to an antibody pathway of response as suggested previously [10]. Additions of glycoarchaeols to PGP archaeosomes enhance greatly the MHC class I pathway of antigen presentation producing the CD8^+^ T cell response. Combination of gentiotriosyl- and mannotriosylarchaeols in the archaeosome adjuvant enhanced the CD8^+^ T cell response over either alone, and the additional presence of archaetidylserine was of further benefit. Finally, long-term immunity was obtained in an archaeol-based lipid archaeosome lacking caldarchaeols. We conclude that for a cancer or intracellular pathogen vaccine where a CD8^+^ T cell response is needed, a favorable archaeosome composition is gentiotriosylarchaeol, mannotriosylarchaeol, AS, and PGP in mol% ratio 22.5/22.5/30/25.

## Supplementary Material

A modified H-phosphonate approach was developed for the synthesis of archaetidylserine. Details of the method and compound characterization are described in this supplementary section.

## Figures and Tables

**Table 1:** Characterization of OVA-archaeosomes.

Archaeosome lipids	Average diameter (nm)	OVA content (*μ*g/mg)
Gentiobiosyl-A/PGP	153 ± 54	19.6
Gentiotriosyl-A/PGP	90 ± 52	21.1
Mannotriosyl-A/PGP	76 ± 41	16.9
Maltotriosyl-A/PGP	97 ± 46	14.7
PGP	92 ± 54	14.2
*M. smithii*	88 ± 51	12.0
