# Supplementary material for: Synthetic Archaeosome Vaccines Containing Triglycosylarchaeols Can Provide Additive and Long-Lasting Immune Responses That Are Enhanced by Archaetidylserine
Source: Archaea. 2012 Sep 30;2012:513231. doi: 10.1155/2012/513231 (PMC3465877; doi:10.1155/2012/513231)
Supplement: Supplementary file 1 — A modified H-phosphonate approach was developed for the synthesis of archaetidylserine. Details of the method and compound characterization are described in this supplementary section. [file 513231.f1.doc]

**Supplementary Materials**

**Summary**

A modified H-phosphonate approach was developed for the synthesis of archaetidylserine. Details of the method and compound characterization are described in this supplementary section.

**General methods**

The ^1^H NMR spectra were obtained on a Varian-400 (400 MHz) with tetramethylsilane or the residual signal of the solvent as the internal standard. The ^13^C NMR spectra were recorded at 100 MHz with a Varian-400 spectrometer. All solvents and reagents were purified and dried according to standard procedures.

**Synthesis of Archaetidylserine**

The following modified H-phosphonate approach was based on Yan and Jenning's procedure [1].

Ammonium 4-methylphenyl H-phosphonate [2] (**1**, 345 mg, 1.82 mmol), methanol (2 mL) and triethylamine (544 μL, 3.90 mmol) were co-evaporated under reduced pressure. The resulting residue was co-evaporated under reduced pressure with pyridine (3 × 2 mL) followed by addition of commercially available Z-Ser-OBzl (200 mg, 0.607 mmol) and pyridine (3.5 mL). Cooled to -20 ºC, Pivotoyl chloride (224  L, 1.82 mmol) was added dropwise over ~ 2 min and stirring continued at -20 ºC under Ar. After 1 h, water (1 mL) was added and the resulting mixture was stirred at room temperature for 2 h. The reaction mixture was concentrated under reduced pressure followed by extraction with CH_2_Cl_2_ and water using a separatory funnel. The bottom organic phase was washed twice with triethylammonium phosphate buffer^1^ [3], dried with MgSO_4_, filtered and concentrated under reduced pressure. The residue was redissolved in pyridine (2 mL) followed by addition of potassium phosphate buffer^2^ (1 M, pH 7, 40 mL) [2]. The resulting solution was stirred at 60 ºC for 5 h followed by workup as above. The desired product **2** was purified by reversed phase (C18) silica gel flash chromatography. Approximately 10 g of reversed phase (C18) silica gel was used. Column conditioning: 1) MeOH wash (2 × 40 mL) 2) 8:2 MeOH/water wash (40 mL) 3) 1:1 MeOH/water wash (40 mL) 4) 2:8 MeOH/water wash (40 mL) 5) water wash (2 × 40 mL). The worked-up residue was loaded with minimum amount of water as a cloudy mixture. Fraction collection (40 mL for each fraction): Fractions 1 & 2 eluted with water; Fractions 3 – 10 eluted with 2:8 MeOH/water; Fractions 11 – 13 eluted with 1:1 MeOH/water. The desired compound **2** was found in Fractions 4 – 10 according to ^1^H NMR spectroscopy. ^1^H NMR (CDCl_3_): 7.47 (m, 1H, ArH), 7.30 (m, 9H, ArH), 6.79 (d, 1H, J_P,H_ = 632, P-H), 5.17 (s, 2H, Z-CH_2_), 5.09 (q, 2H, Bn-CH_2_), 4.44 (m, 1H, Hα), 4.30 (m, 1H, Hβ), 4.20 (m, 1H, Hβ'), 2.94 (brq, 6H, Et_3_N-CH_2_), 1.20 (brt, 9H, Et_3_N-CH_3_). This process yielded **2** as a clear colourless oil (197 mg, 65.8 %).

Compound **2** (123.7 mg, 0.25 mmol) and archaeol^3^ [4] (147 mg, 0.22 mmol) were co-evaporated with pyridine (2 × 1.5 mL) and the resulting residue was redissolved in pyridine (4.5 mL), cooled to –5 ºC followed by dropwise addition of pivotoyl chloride (77 μL, 0.62 mmol) over ~ 2 min. The mixture was stirred at –5 ºC under Ar for 30 min and then quenched by adding water (6 mL). Stirring continued at room temperature for another 30 min followed by solvent evaporation. The resulting residue was then dissolved in CH_2_Cl_2_, washed with saturated aqueous NaHCO_3_ (2 ×), washed with triethylammonium phosphate buffer^1^ (2 ×), washed once with water, dried with MgSO_4_, filtered and concentrated under reduced pressure. The crude desired product **3** was carried on to the next step without purification. Partial ^1^H NMR (CDCl_3_): 6.83 (d, 0.5H, J_P,H_ = 723, P-H), 6.74 (d, 0.5H, J_P,H_ = 619, P-H). 5.22 (brs, 2H, Z-CH_2_), 5.13 (q, 2H, Bn-CH_2_).

Compound **3** (227.5 mg, 0.221 mmol) was dissolved in pyridine/water (4:1 v/v, 3.5 mL) followed by addition of I_2_ crystals (40 mg, 0.158 mmol). The resulting brown solution was stirred at room temperature for 1 h. Aqueous Na_2_S_2_O_3_ (0.1 M, 3.5 mL) was added followed by extraction with CH_2_Cl_2_ (3 × 8 mL). The organic phase was washed with triethylammonium phosphate buffer^1^ (2 × 5 mL), washed once with water, dried with MgSO_4_, filtered and concentrated under reduced pressure. The brown crude residue was purified on a LH 20 column to obtain compound **4** (188 mg, 85 %). [Column size: 60 × 2.5 cm; Solvent: 4:3 CHCl_3_/MeOH; Collection: 5 min/fraction; Time: ~ 1 ½ h after loading (fractions 20-24)] ^1^H NMR (CDCl_3_): 7.34-7.26 (m, 10H, ArH), 5.18 (q, 2H, Z-CH_2_), 5.08 (q, 2H, Bn-CH_2_), 4.43 (m, 1H, Hα), 4.34 (m, 1H, Hβ), 4.26 (m, 1H, Hβ'), 3.87 (m, 2H, arch-aa'), 3.58 (m, 4H, arch-b, arch-c, arch-ee'), 3.42 (m, 3H, arch-c', arch-dd'), 2.93 (brq, 6H, Et_3_N-CH_2_), 1.62-1.36 (m, 6H, arch-CH,CH_2_), 1.34-1.02 (51H, Et_3_N-CH_3_, arch-CH,CH_2_), 0.86 (m, 30H, arch-CH_3_). ^13^C NMR (CDCl_3_): 169.8 (C=O-O), 168.7 (C=O-N), 156.4 (Z-C=O), 136.6 (Ar*_ip_*), 135.6 (Ar*_ip_*), 128.6-127.6 (Ar-CH), 77.8 (acrh-b), 70.7 (arch-c), 70.0 (arch-e), 68.7 (arch-d), 67.0 (Z-CH_2_), 66.6 (Bn-CH_2_), 65.2 (br, arch-a, Cβ), 55.7 (Cα), 45.4 (Et_3_N-CH_2_), 39.3, 37.55, 37.50, 37.43, 37.40, 37.3, 37.0, 36.7 (arch-CH_2_), 32.8, 29.9, 29.8, 27.9 ( arch-CH), 24.8, 24.4, 24.3 (arch-CH_2_), 22.7, 22.6, 19.7, 19.6 (arch-CH_3_), 8.4 (Et_3_N-CH_3_).

Archaetidylserine (**5**)

Compound **4** (187 mg, 0.163 mmol) was dissolved in 1:1 EtOAc/MeOH (12 mL) and was hydrogenated at 50 psi at room temperature in the presence of a catalytic amount (~ 190 mg) of 10 % Pd/C for 16 h. The resulting mixture was filtered through a bed of Celite followed by solvent evaporation under reduced pressure. The lipid was extracted [5] from the residue which was redissolved in 12.5:25:10 CHCl_3_/MeOH/EDTA buffer^4^ (total volume used = 47.5 mL) and the resulting one-phase solution was stirred at room temperature for 3 h. CHCl_3_ (11 mL) and EDTA buffer^4^ (11 mL) were then added to recover the lipid in the lower CHCl_3_ phase using a separatory funnel. The upper phase was further extracted with CHCl_3_ (2 × 11 mL) and the combined CHCl_3_ phase was passed through a piece of filter paper followed by solvent evaporation under reduced pressure. The residue was redissolved in CHCl_3_ and then was washed three times with 0.01 M aqueous NaHCO_3_ (pH ~ 8) and the aqueous phase was back extracted with CHCl_3_. Solvent removal of the combined organic phase yielded the desired compound **5** (84 mg, 61 %). ^1^H and ^13^C NMR spectra were obtained in 1:1 CDCl_3_:CD_3_OD and ESI-positive ion MS was obtained with addition of formic acid. The identity was further checked by TLC (85:22.5:10:4 CHCl_3_/CH_3_OH/AcOH/H_2_O) which along with the spectral data are consistent with archaetidylserine purified from natural sources^3^ [4;6], where parameters were [α]_D_ 0.7° (*c*, 0.010, CHCl_3_); ^1^H NMR (CDCl_3_:CD_3_OD 1:1): 4.28 (ddd, 1H, J_β,β'_ = 12.0, J_α,β_ = 3, J_H,P_ = 7.4, H-β), 4.20 (ddd, 1H, J_α,β'_ = 6.4, H-β'), 4.02 (dd, 1H, Hα), 3.86 (m, 2H, arch-aa'), 3.62 (m, 3H, arch-dd', arch-b), 3.56 (dd, 1H, J_c,c'_ = 10.4, J_b,c_ = 3.6, arch-c), 3.48 (m, 3H, arch-c', arch-ee'), 1.62-1.45 (m, 6H, CH, CH_2_), 1.40-1.00 (m, 42H, CH, CH_2_), 0.99-0.82 (m, 30H, CH_3_). ^13^C NMR (CDCl_3_:CD_3_OD 1:1): 78.5 (arch-b), 71.4 (arch-c), 70.8 (arch-d), 69.6 (arch-e), 65.8 (J_C,P_ = 6.4, Cβ), 63.6 (arch-a), 54.6 (Cα), 40,1, 38.2, 38.12, 38.08, 38.0, 37.7, 37.6 (CH_2_), 33.5, 30.6, 30.5, 28.7 (CH), 25.5, 25.1 (CH_2_), 23.1, 23.0, 20.23, 20.21, 20.1 (CH_3_). ESI +ve M+H 820.5; ESI -ve M-H (818.7).

**Footnotes**

^1^ Preparation of triethylammonium phosphate buffer: A 0.5 M H_3_PO_4_ solution of which the pH was adjusted to 7 to 8 according to pH paper by adding Et_3_N.

^2^ Preparation of potassium phosphate buffer: 1) Monobasic KH_2_PO_4_ (27.22 g) was dissolved in water (200 mL) to generate a 1 M solution of monobasic stock (pH ~ 5). 2) Dibasic K_2_HPO_4_ (17.42 g) was dissolved in water (100 mL) to generate a 1 M solution of dibasic stock (pH ~ 9). 3) The monobasic stock was added to the dibasic stock until pH reached ~ 7 by pH paper.

^3^ Observed [α]_D_ 9.1° (*c*, 0.006, CHCl_3_) was consistent with the [α]_D_ 8.5° (*c*, 0.006, CHCl_3_) reported in Morii and Koga [4].

^4^ Preparation of EDTA buffer: Sodium acetate (0.82 g) was dissolved in deionized water (100 mL) followed by addition of ~ 20 drops of AcOH to generate a 0.1 M sodium acetate solution (pH ~ 5 by pH paper). To the sodium acetate solution was added EDTA (acid free, 29 mg) to obtain an EDTA concentration of 1 mM.

Bibliography

[1] H. Yan and H. J. Jennings, "Synthesis of mono- and di-sialophospholipids via the H-phosphonate approach.," *Can. J. Chem.*, vol. 84, pp. 540-545, 2006.

[2] V. Ozola, C. B. Reese, and S. Quanlai, " Use of ammonium aryl H-phosphonates in the preparation of nucleoside H-phosphonate building blocks.," *Tetrahedron Letters*, vol. 37, pp. 8621-8624, 1996.

[3] H. Lindner, B. Sarg, B. Hoertnagl, and W. Helliger, "The microheterogeneity of the mammalian H1(0) histone. Evidence for an age-dependent deamidation," *J. Biol. Chem.*, vol. 273, no. 21, pp. 13324-13330, May1998.

[4] H. Morii and Y. Koga, "Absolute stereochemical configuration of a diphytanyl ether analog of phosphatidylserine of *Methanobrevibacter arboriphilus*,"*,* 879 ed 1986, pp. 103-105.

[5] G. D. Sprott, G. Ferrante, and I. Ekiel, "Tetraether lipids of *Methanospirillum hungatei* with head groups consisting of phospho-N,N-dimethylaminopentanetetrol, phospho-N,N,N-trimethylaminopentanetetrol, and carbohydrates," *Biochim. Biophys. Acta*, vol. 1214, no. 3, pp. 234-242, Oct.1994.

[6] G. D. Sprott, J. Brisson, C. J. Dicaire, A. K. Pelletier, L. A. Deschatelets, L. Krishnan, and G. B. Patel, "A structural comparison of the total polar lipids from the human archaea *Methanobrevibacter smithii* and *Methanosphaera stadtmanae* and its relevance to the adjuvant activities of their liposomes," *Biochim. Biophys. Acta*, vol. 1440, no. 2-3, pp. 275-288, Sept.1999.
